# Primary autoimmune myelofibrosis: A case report in a child

**DOI:** 10.1002/jha2.38

**Published:** 2020-06-28

**Authors:** Zufit Hexner‐Erlichman, Joanne Yacobovich, Philippe Trougouboff, Moran Avraham‐Kelbert, Harel Eitam, Ronen Spiegel, Shay Yeganeh, Carina Levin

**Affiliations:** ^1^ Pediatrics Department “B” Emek Medical Center Afula Israel; ^2^ Department of Pediatric Hematology Oncology Schneider Children's Medical Center of Israel Petach Tikva Israel; ^3^ Sackler School of Medicine Tel Aviv University Tel Aviv Israel; ^4^ Pathology Department Emek Medical Center Afula Israel; ^5^ Technion–Israel Institute of Technology Haifa Israel; ^6^ Pediatric Hematology Unit Emek Medical Center Afula Israel; ^7^ Baruch Padeh Medical Center, Poriya Haifa Israel; ^8^ Bar Ilan Medical School Bar Ilan University Ramat Gan Israel

**Keywords:** anemia, autoimmunity, lymphopenia, myelofibrosis

## Abstract

Autoimmune myelofibrosis (AIMF) is an uncommon cause of myelofibrosis associated with favorable outcome. Primary AIMF, AIMF without a known systemic autoimmune disorder, has been described in adults, but never in children. Here, we present, for the first time, an apparent case of primary AIMF in a 15‐year‐old boy admitted with profound hypoproliferative anemia.

## Abstract 2

For the first time, we describe a pediatric case of primary autoimmune myelofibrosis presented to the emergency room with profound hypoproliferative, low reticulocyte count, anemia.

**Abbreviations**
AIMF: autoimmune myelofibrosis
BM: bone marrow
BMF: bone marrow fibrosis
CBC: complete blood count
CD: cluster of differentiation
MDS: myelodysplastic syndrome
MF: myelofibrosis
PBS: peripheral blood smear
PMF: primary myelofibrosis

## 1 INTRODUCTION

Autoimmune myelofibrosis (AIMF) is a rare entity that manifests by autoimmune phenomena, bone marrow fibrosis (BMF), cytopenias, and minimal or no splenomegaly. It is classified into primary AIMF when autoantibodies are found in the absence of a known systemic autoimmune disorder and secondary when is associated with an established autoimmune disease. The pathophysiology of AIMF is poorly understood, however, immune dysregulation is known to play a pivotal role. Hence, the treatment of choice is immunosuppressive therapy.

## 2 CASE REPORT

A 15‐year‐old boy presented to his general practitioner in August 2018 complaining of headaches that worsened with physical activity and severe pallor was observed. A complete blood count (CBC) revels; normocytic anemia (hemoglobin = 5.9 g/dL, mean corpuscular volume [MCV] = 78 fL, and 1% of reticulocyte) and the patient was referred to his local hospital.

The patient's previous medical history included bilateral sensorineural and conductive hearing loss diagnosed during childhood, rheumatological evaluation, and follow‐up due to joint and abdominal pain. Heterozygosity for Familial Mediterranean Fever (E148Q mutation) was the only finding; empiric treatment with colchicine was administered for 6 years and discontinued by the patient 1 month before presentation. Microcytic anemia and lymphopenia were documented in the 3 years prior to presentation. The boy's family history for diseases was unremarkable. Physical examination upon the patient's arrival at the hospital was normal, except for severe pallor.

The patient's initial laboratory assessment is shown in Table 1. The CBC showed leukopenia with lymphopenia, severe normocytic anemia with low reticulocytes, and a normal platelet count. The peripheral blood smear (PBS) demonstrated some ovalocytes, poikilocytosis, and anisocytosis. Iron, ferritin, transferrin, folic acid, vitamin B12, electrolytes, lactate dehydrogenase (LDH), bilirubin, and C‐reactive protein levels were slightly elevated. Renal, liver, and thyroid function were normal. Direct antiglobulin test was negative. A mildly enlarged spleen (14 cm span) and mild mesenteric lymphadenopathy were documented by abdominal ultrasound and computed tomography. At this point, the patient was transfused with a unit of packed red blood cells and referred to Emek Medical Center for further assessment.

**TABLE 1:** Laboratory findings at diagnosis, during therapy, and at recovery

12 mo	8 mo	3 mo	3 wk	Diagnosis	Laboratory parameter
6.63	5.01	5.34	9.98	3	White blood cell count, 10^3^/μL
1.440	3.34	1.110	880	960	Lymphocyte absolute count, 10^3^/μL
11.6	11.9	12.3	13.4	5.9	Hemoglobin, g/dL
81	85.5	74.4	92.8	78.4	MCV, fL
15	15.8	15.5	18.9	20	Red cell distribution width (RDW), %
1.6	1.5	1	2.1	1	Reticulocyte, %
306	321	329	191	329	Platelet count, 10^3^/μL
				Negative	Direct antiglobulin test
	Normal			Normal	Iron, ferritin, transferrin, folic acid, and vitamin B12
	0.7			0.54	CRP (mg/dL), NV 0.0‐0.5
	Normal			Normal	Thyroid function
	Normal			Normal	RFTs (serum electrolytes)
	Normal			Normal	LDH (U/L), bilirubin (mg/dL), LFTs
				Negative	Serology for EBV, CMV, HBV, HCV, and brucellosis
				Negative	PCR for Parvovirus B19
					Immunoglobulins
	985			1228	‐ IgG (mg/dL), NV 600–1400
	43			63	‐ IgA (mg/dL), NV 90–430
	91			34	‐ IgM (mg/dL), NV 33–200
	Normal			Normal	Complement levels (mg/dL) C3, C4
	Negative (titer 1:80)			Negative (titer 1:80)	Antinuclear antibody pattern
				2437	Erythropoietin (mIU/mL), NV 4–29
	Negative			Negative	Rheumatologic antibody panel, AI. (RNP, anti‐Smith Ab, SSA, SSB, RNP A, RNP 68, Sm/RNP, SSA‐60, SSA‐52, chromatin, Anti Jo‐1 Ab, Scl‐70 Ab, anti‐centromere Ab, ribosomal P Ab)
	21			20	dsDNA antibody (IU/mL), NV 0.0–5.0
				Slightly positive (titer 1:40)	Smooth muscle antibody, immunofluorescence
				Negative	Anti‐myeloperoxidase, AI
				Negative	Anti‐proteinase 3, AI

Abbreviations: NV, normal values; wk, week; mo, month; MCV, mean corpuscular volume; CRP, C‐reactive protein; RFTs, renal function tests; LDH, lactate dehydrogenase; LFTs, liver function tests; EBV, Epstein‐Barr virus; CMV, cytomegalovirus; HBV, hepatitis B virus; HCV, hepatitis C virus; RNP, ribonucleoprotein; SSA, anti‐Sjögren's syndrome type A; ab, antibody; SSB, anti‐Sjögren's syndrome type B; Sm/RNP, smith/ribonucleoprotein; Scl‐70 Ab, scleroderma 70 antibody; AI, antibody index; dsDNA, double‐stranded DNA antibody.

**FIGURE 1:**
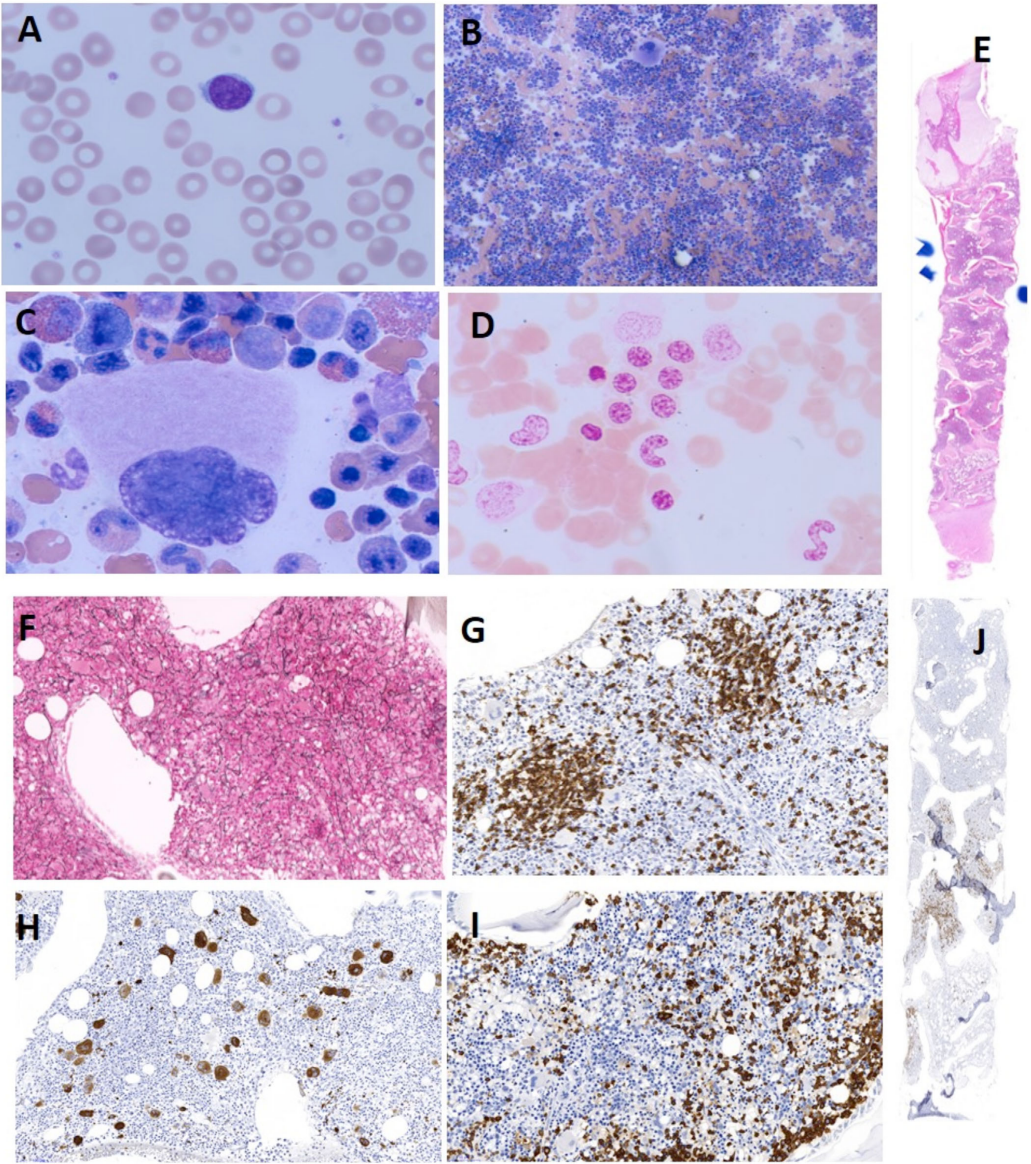
Peripheral blood smear and bone marrow. A, PBS (Wright's stain; ×1000). B‐D, BM aspirate; B, (×100); C, (×1000) hypercellularity, trilineage maturation; D, (×1000) iron stain. E‐J, BM biopsy; E, hypercellularity; F, reticulin staining (MF 1–2); G, CD3‐positive lymphocyte; H, CD61‐positive megakaryocytes; I, myeloperoxidase staining; J, CD71‐positive erythropoietic precursors

The 15‐year‐old boy presented with symptomatic hyporegenerative anemia, requiring three units of packed red blood cells during the first week of hospitalization. The PBS demonstrated some ovalocytes, poikilocytosis, and anisocytosis; no nucleated red blood cells or tear drops are observed (Figure 1, Panel A). An extensive laboratory workup was performed and was essentially negative for infectious and rheumatoid diseases, except for positive serum double stranded (ds) DNA and slight positive smooth muscle antibodies. Erythropoietin levels were markedly elevated (Table 1). Peripheral blood immunophenotyping was negative for paroxysmal nocturnal hemoglobinuria, and genotyping for the *JAK2* V617F mutation was negative. Bone marrow (BM) aspiration (Figure 1, Panels B‐D) demonstrated hypercellularity, trilineage maturation with mild megaloblastic, and dysplastic changes in the erythroid and myeloid progenitors, with no presence of sideroblasts in iron stain. The BM biopsy (Figure 1, Panels E‐J) showed hypercellular BM (up to 90%), with slight megakaryocytic and erythroid dysplastic changes and diffuse to focally moderate reticulin myelofibrosis (MF1–2) with foci of paratrabecular fibroblastic and vascular proliferation. Trichrome collagen stain was negative. Along the BM, there were several prominent lymphohistiocytic aggregates (CD4‐positive alpha‐beta T cells, where CD is cluster of differentiation), associated with increased interstitial small lymphocytic infiltration (CD8‐inactivated cytotoxic T cells [TIA1‐positive, granzyme‐B negative]), with no evidence of T‐cell lymphoproliferative disease or myeloproliferative neoplasm. These features are compatible with reactive changes due to autoimmune disease. BM immunophenotyping indicated increased myelopoiesis with no monoclonality and no increase in blasts. Cytogenetic and fluorescence in situ hybridization panel (see Supporting Information Data) for eight known changes linked to myelodysplastic syndrome (MDS)/acute myelogenous leukemia was negative. The BM genetic panel, which included sequences of *JAK2*, *CALR*, and *MPL* (and additional 49 genes, see Supporting Information Data), did not reveal any pathological variants.

Given a presumptive diagnosis of primary AIMF, the patient was treated with 60 mg prednisone (∼1 mg/kg) twice daily for 21 days. The dose was slowly tapered off over 3 months.

Within the first weeks of therapy, reticulocytosis appeared and hemoglobin level increased with no further need for transfusions. The patient developed new signs and symptoms of arthritis, thus a thorough rheumatological workup was performed. The previous diagnosis of familial Mediterranean fever was reconsidered, but no specific diagnosis was established, although colchicine treatment was not reinstated. The patient continues to be under close follow‐up to ensure the maintenance of his hematological response.

## 3 DISCUSSION

Hypoproliferative or central origin anemia, characterized by an inappropriately low reticulocyte count, comprises a heterogeneous group of anemias caused by the BM's inability to produce adequate numbers of red blood cells. After initial evaluation of the most common causes, including nutritional deficiencies, and exclusion of endocrinological, autoimmune, infectious, and chronic diseases, specific tests and BM examination are indicated. Additional differential diagnoses include leukemia, MDS, BM failure (congenital or acquired), myelofibrosis, or other malignant or pathological BM infiltration [1].

BMF can be secondary to a wide range of conditions, from BM metastases/neoplasms or MDS to nonneoplastic entities such as infections, endocrine disorders, or autoimmune disease. The most common cause of BMF in adults is primary myelofibrosis (PMF). PMF patients present with splenomegaly, anemia, and a peripheral blood smear showing immature red and white blood cells, teardrop red cells, and high serum LDH. Clonality and mutations in *JAK2*, *MPL*, or *CALR* are found in 90% of the cases. It is extremely important to differentiate AIMF from PMF, since the clinical course, prognosis, and treatment are vastly different [2, 3].

AIMF is a benign and rare entity characterized by cytopenias, autoimmune phenomena, BMF, and minimal or no splenomegaly [4, 5]. Primary AIMF refers to patients that present autoantibodies in the absence of an identifiable systemic autoimmune disorder [4]. When AIMF is found in association with systemic lupus erythematosus or other established autoimmune conditions, it is termed secondary AIMF. No pediatric cases of primary AIMF have been reported; however, several adult cases have been published [6, 7]. In 2003, primary AIMF in adults was defined by a set of diagnostic criteria: (1) grade 3–4 reticulin fibrosis of the BM; (2) lack of clustered or atypical megakaryocytes; (3) lack of myeloid or erythroid dysplasia, eosinophilia, or basophilia; (4) lymphocyte infiltration of the BM; (5) lack of osteosclerosis; (6) absent or mild splenomegaly; (7) presence of autoantibodies; and (8) absence of a disorder known to cause myelofibrosis [8, 9].

Although the pathophysiology of AIMF is poorly understood, aberrant cytokine production has been described as having a pivotal role. Harrison and colleagues [10] reported increased serum levels of transforming growth factor beta and substance P in an AIMF patient, which decreased following treatment. This suggested a role for immune dysregulation, including T‐cell dysfunction, in the pathogenesis of AIMF.

The differential diagnosis in our patient included underlying immune‐related disease suggested by increased BM cellularity and slight positive autoimmune serology. Negative Direct Coombs Test and reticulocytopenia reduced the likelihood of autoimmune hemolytic anemia. Our patient did not display characteristics of PMF: no splenomegaly, the peripheral blood smear showed no teardrop or immature erythrocytes, or basophilia or eosinophilia. The rheumatological versus autoimmune systemic diagnoses in our patient remains nonspecific and inconclusive, supporting the hematological diagnosis of primary AIMF. Despite the rareness of this condition, we considered the possibility that it is the cause of our patient's severe anemia.

Our patient did not fulfill all the pathological diagnostic criteria for primary AIMF published for adults. The BM biopsy showed some erythroid and megakaryocytic dysplasia, and only grade 1–2 fibrosis (in itself an uncommon finding in pediatric BM). Despite these discrepancies, we did not rule out this condition since the clinical and laboratory picture of AIMF has not been described in the pediatric realm. Primary AIMF was previously reported as a condition mimicking MDS [11], we considered this diagnosis as well. Our patient's anemia, lymphopenia, and the presence of increased BM cellularity, accompanied by fibrosis with mild megakaryocytic and erythroid dysplasia, raised the suspicion of hypercellular MDS (a rare form of MDS in children). However, the absence of clonal, cytogenetic, or germline abnormalities associated with MDS, together with positive autoimmune serology results, made this diagnosis less likely. Importantly, after 1 year of follow‐up, the patient continues to be in complete remission after being treated solely with glucocorticosteroids.

Immunosuppressive agents, especially prednisone, are the main treatment for primary AIMF with rapid improvement in most cases [6, 7, 8], as in ours case. Patients who fail to respond to corticosteroids may benefit from other immunosuppressive modalities. Despite recovery from cytopenia, in some cases BMF does not resolve, suggesting that the pathogenesis independently leads to altered hematopoiesis, in addition to induction of fibrosis [8].

To the best of our knowledge, no case reports of presumed primary AIMF in children have been published, and therefore, the long‐term prognosis of these patients remains unknown.

In conclusion, primary AIMF is a rare disease in adults; the present case study appears to be the first description of a pediatric case. This condition requires meticulous clinical assessment, including a high degree of suspicion of autoimmune etiology. Additional studies are needed to shed more light on the effect of aberrant cytokine production on primary AIMF, and define its clinical picture and natural history, particularly in children.

## CONFLICT OF INTEREST

The authors declare that there is no conflict of interest.

## CONSENT TO PUBLISH

The participant's parents have consented to the submission of the case report to the journal.

## DATA SHARING STATEMENT

We shall share information concerning patient's history, physical examination, imaging studies, and laboratory studies. The appropriate consent to participate and publish has been described as well.

## Supporting information

Supporting Information
