# Supplementary material for: Primary autoimmune myelofibrosis: A case report in a child
Source: EJHaem. 2020 Jun 28;1(1):304–8. doi: 10.1002/jha2.38 (PMC9175846; doi:10.1002/jha2.38)
Supplement: Supplementary file 1 — Supporting Information [file JHA2-1-304-s001.doc]

**Supplementary Appendix**

**Table of Contents**

Laboratory Studies 3

Bone Marrow (BM) Aspiration 4-5

BM Biopsy 6

**Laboratory Studies**

Blood work was performed at Emek Medical Center.

Complete blood count (CBC), including reticulocytes, was obtained using an ADVIA 2120i analyzer (Siemens, Germany).

Iron, transferrin, C-reactive protein (CRP), blood urea nitrogen, creatinine, electrolyte, lactate dehydrogenase (LDH) and bilirubin levels and liver function tests were determined using an Olympus AUS 800 analyzer (Beckman Coulter, USA).

Ferritin, folic acid and vitamin B12 levels, as well as thyroid function, were measured with an ADVIA Centaur XP analyzer (Siemens).

Erythropoietin level was measured by the Immulite 2000XPi (Siemens).

Immunoglobulin and complement levels, and serology test for hepatitis B and C were examined with the Cobas 6000 analyzer (Roche Diagnostics, USA). Brucellosis test was performed with a Rose Bengal kit (Bio-Rad, USA). Serology tests for Epstein–Barr virus and cytomegalovirus were examined using the Architect i2000 analyzer (Abbott, USA).

Rheumatologic antibody panel, as well as levels of antinuclear antibody, double-stranded DNA antibody, anti-myeloperoxidase and anti-proteinase 3 antibodies were examined using the Bioplex 2200 using multiplex flow immunoassay (Bio-Rad Laboratories, Inc, CA, USA). Antinuclear antibodies were also tested by immunofluorescence using the HELIOS analyzer (Aesku Diagnostics GmbH & Co. KG, Wendelsheim, Germany). dsDNA IgG was also tested by ELISA (Aesku Diagnostics). Smooth muscle cell antibodies were tested by immunofluorescence using Kallestad Mouse stomach/ kidney slides (Bio-Rad Laboratories).

**Bone Marrow (BM) Aspiration**

Immunophenotyping was performed using flow cytometry at Emek Medical Center. Approximately 5 x 10^6^ cells from EDTA (Ethylenediaminetetraacetic acid)-anticoagulated BM were stained with monoclonal antibodies as used in the eight-color BM panel: CD45, CD34, CD71, CD10, CD64, CD33, CD19, CD20, kappa and lambda light chains, CD3, CD4, and CD8. The cells were incubated for 10 min at room temperature in the dark. The erythrocytes were lysed with commercial lysing reagent BD FACS^TM^ Lysing Solution (Becton Dickinson, USA) for 10 min in the dark. The sample was centrifuged for 5 min at 300*g*. The pellet was resuspended in 1 mL phosphate buffered saline (1X PBS). The sample was centrifuged again for 5 min at 300*g*, the supernatant was decanted and the cells were resuspended in 500 µL PBS. Fluorescence emission was read in a NAVIOS flow cytometry machine (Beckman Coulter). Cell population identification was based on forward-scatter and side-scatter parameters and CD45/side-scatter gating methods. A minimum of 10,000 events were gated for analysis. Flow cytometry list mode files were analyzed with NAVIOS and KALUZA software.

Cytogenetic analysis was performed at Schneider Children's Medical Center.

BM cells were processed after 24 h in unstimulated culture (RPMI 1640 medium, 20% concentration fetal calf serum). A routine technique was followed, including Colcemid, hypotonic KCl solution, fixation in methanol/acetic acid and trypsin-Giemsa for G-band staining. Twenty metaphase chromosomes were analyzed and their karyotype defined according to the International System for Human Nomenclature (2019).

Fluorescence in situ hybridization (FISH) was performed at Schneider Children's Medical Center using the Chromoprobe Multiprobe AML/MDS cytocell panel, which has eight directly labeled FISH probes applied to a single slide performed in Hybrite device. A BM sample of suitable concentration was dropped onto the corresponding template, using only 4 µL per probe to be hybridized. After denaturation at 72^o^C for 2 min and overnight hybridization at 37^o^C, unbound and nonspecifically bound DNA probes were removed by two different stringent washes: 0.4X SSC (Saline-sodium citrate buffer) at 72^o^C for 2 min and then 2X SSC, 0.05% Tween 20 at room temperature for 30 seconds. Then the slide was counterstained with DAPI (4′,6-Diamidine-2′-phenylindole dihydrochloride) for visualization under a microscope. This panel detects rearrangements such as: del/monosomy (5q), del/monosomy (7q), iso(17q), trisomy 8, and trisomy 21 (through ETO/AML1, also designed for the 8;21 translocation), del(20q), inv(16) and PML/RARA fusion (15;17) in a single experiment.

Targeted panel next generation sequencing (NGS) analysis was performed at Schneider Children's Medical Center for suspected BM failure syndromes or myelodysplastic syndromes (MDS). Targeted NGS sequencing involves isolation of genomic regions of interest. After fragmenting the genome, the desired fragments are captured by hybridizing to baited probes, which are then isolated. A standard library is then prepared for NGS. The captured libraries are currently being sequenced on an Illumina MiSeq system (at the Hebrew University of Jerusalem) with 300 bp paired-end reads with minimum coverage of 200X over 95% of the target regions. The proband’s DNA sequences are mapped and compared to the human genome reference sequence (GRCh37). Upon receiving sequence results, assessment of coverage and quality for targeted coding exons of the known protein-encoding RefSeq genes is performed. Common variants in populations (public databases and internal database) and variants without clear functional effect (intronic, intergenic and synonymous) are filtered out. Sanger sequencing is performed on the suspected variants for validation.

List of genes: *ASXL1, ATRX, BCOR, BRAF, CALR, CBL, CDKN2A, CEBPA, CREBBP, CTNNA1, CUX1, DNMT3A, EP300, ETV6, EZH2, FIZ1, FLT3, GATA2, GNAS, IDH1, IDH2, IRF1, JAK2, KDM6A, KIT, KRAS, MYD88, MLL2, MPL, NF1, NPM1, NRAS, PHF6, PPM1D, PTEN, PTPN11, RAD21, RUNX1, SAMD9, SAMD9L, SF3B1, SFRS2, SH283, SMC1A, SMC3, SRSF2, SUZ12, TET2, TP53, U2AF1, WT1, ZRSR2.*

**BM biopsy**

BM biopsies were studied at Emek Medical Center. Formalin-fixed paraffin-embedded (FFPE) biopsy specimen sections (3 µm) were stained with hematoxylin and eosin (Leica ST5020, Heidelberger, Germany). All specimens were decalcified in a 10% EDTA solution (pH 7.2–7.4). Next, the specimens were processed in an automated immunostainer (BenchMark Ultra; Ventana Medical Systems, USA) and stained with antibodies against CD61, CD71, CD34, TdT, CD117, CD25, CD14, CD163, CD68, CD3, CD5, CD2, TCR-BF-1 (alpha-beta), TCRD (gamma-delta), TIA-1, Granzyme B, CD56, CD20, and CD38. All antibodies were evaluated against internal or external controls. Histochemical stains for reticulin, Prussian blue and Congo red, were processed from 5-µm FFPE biopsy specimen sections using an automated special stainer (BenchMark; Ventana Medical Systems).
